# Time-resolved photon counting Fourier-transform micro-spectroscopy enables simultaneous Raman and fluorescence lifetime imaging

**DOI:** 10.1038/s41377-025-02020-8

**Published:** 2025-10-29

**Authors:** Lindong Shang, Xiaodong Bao, Hao Peng, Fuyuan Chen, Yu Wang, Kunxiang Liu, Peng Liang, Yuntong Wang, Xusheng Tang, Francesco Masia, Wolfgang Langbein, Bei Li

**Affiliations:** 1 Changchun Institute of Optics, Fine Mechanics and Physics, Chinese Academy of Sciences, Changchun, 130033 China; 2 University of Chinese Academy of Sciences, Beijing, 100049 China; 3 State Key Laboratory of Advanced Manufacturing for Optical Systems, Chinese Academy of Sciences, Changchun, 130033 China; 4 Shenzhen Institute of Advanced Research, University of Electronic Science and Technology of China, Shenzhen, 518038 China; 5 Hooke Instruments, Changchun, 130033 China; 6 School of Biosciences, Cardiff University, Cardiff, CF10 3AX UK; 7 School of Physics and Astronomy, Cardiff University, Cardiff, CF24 3AA UK

**Keywords:** Raman spectroscopy, Imaging and sensing

## Abstract

To address the current limitations of time-gated Raman spectroscopy, specifically its narrow spectral range and low spectral resolution, and simultaneously acquire Raman and fluorescence life-time images, we have developed a Fourier-transform photon counting spectroscopy platform. A Mach-Zehnder interferometer employing a high accuracy linear motor stage was combined with photon-counting avalanche diodes and time-tagged acquisition, allowing to sort photons into a matrix of stage positions determined using their coarse arrival time with 50 ns steps of the excitation laser repetition period, and a fine arrival time of 80 ps resolution relative to the excitation pulse of 100 ps duration. The instrument achieves a time resolution of 547 ps, a wide spectral range of −1000 to 10,000 cm^−1^ Raman shift from the excitation at 532 nm wavelength, and a high spectral resolution of 0.05 cm^−1^. For experimental validation, we used fluorescently coated silicon wafers and fluorescent plastic microspheres. Raman signal was observed during the laser excitation pulse within the time-resolution, while fluorescence signals dominate afterwards. The results confirm that the instrument can effectively separate Raman and fluorescence signals.

## Abstract (web summary)

This work reports on a new concept of time-resolved photon counting Fourier-transform micro-spectroscopy, allowing continuous scanning by converting photon arrival times to interferometer position.

## Graphical abstract

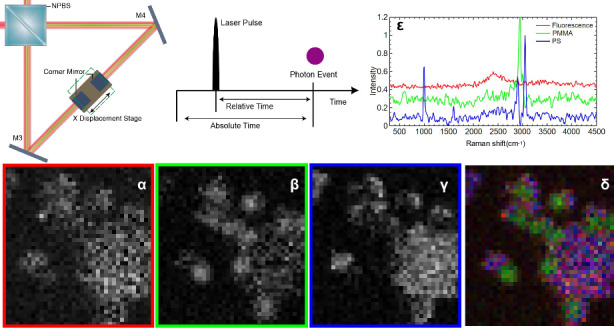

## Introduction

Raman spectroscopy is an important tool for material science^1–4^, semiconductor industry^5,6^, environmental testing^7–9^, archeology^10–12^, and biomedicine^13–16^. One significant limitation is fluorescence background^17,18^, which can overwhelm Raman scattering. Most Raman spectroscopy uses continuous excitation and detection^19,20^, making it impossible to separate Raman and fluorescence components based on their temporal dynamics, with Raman being instantaneous and fluorescence having a typical lifetime in the nanosecond range. Instead, spectral information is often used for separation. Fluorescence has a spectral shift from the excitation given by a sum of many vibrational interactions, both adding and subtracting vibrational energies, leading to a broad continuum of shifts, while Raman is involving only one or sometimes two vibrational interactions, providing discrete shifts. Therefore, it is common practice to subtract from the measured emission spectrum a spectrally broad background, capturing the fluorescence. However, also Raman scattering of dense, spectrally overlapping vibrations is removed. Moreover, the photon shot-noise of the fluorescence background remains, decreasing sensitivity^21^. Furthermore, subtracting such a fitted background independently for the spectrum of each spatial point is prone to create systematic errors^22^.

Separation of Raman and fluorescence using their distinct dynamics is achieved in time-gated Raman, which uses a pulsed laser, and collects the Raman signal within a short time gate, suppressing fluorescence^23^. Time-gated Raman uses either Kerr gates^24^, or gated intensified charge-coupled devices (ICCD)^25^, or single-photon avalanche diode (SPAD) time-resolved photon counting detection^26^. Kerr gates use a third-order nonlinearity created by strong laser pulses, allowing very short gates below a picosecond. The required pulse energies limit the laser repetition rates, and the gate is often of low efficiency^27,28^. ICCDs electrically gate the intensifier providing some 250 ps-1ns resolution and a (1–40%) quantum efficiency (QE) given by the photocathode used^29,30^. SPADs are rapidly developing over the last decade due to demands of quantum optical applications, and are of lower cost^31,32^. Their sensitive surface is small, and their single channel nature prevents the simultaneous detection of spectrally resolved data, so that spectra require either time-sequential spectral scanning, or Fourier-transform techniques^33^. SPAD arrays are presently under development and might present a suitable detector for time-gated Raman in the future^34,35^. Using a Fourier-transform spectrometer, only a single detector is needed since the spectral resolution is achieved by scanning an optical delay, and provides a wider spectral range and controllable and high spectral resolution.

In this work, we have developed a time-gated Raman system based on a high-resolution Fourier-transform spectrometer and SPADs. We designed a photon matrix to store the photon events detected by the SPAD, and we have developed a strategy to correct the obtained photon interference matrix for the measured stage motion, allowing continuous stage scanning. The instrument demonstrates a time resolution of 547 ps (FWHM), with user-adjustable spectral range and resolution capabilities. The system achieves a broad spectral coverage from −1000 to 10,000 cm⁻¹, with a maximum achievable resolution of 0.05 cm⁻¹. To validate the system, we conducted experiments using fluorescently coated silicon wafers and fluorescent microspheres. The results demonstrate that the instrument can effectively separate Raman and fluorescence signals.

## Results

### Time-resolved photon-counting Fourier-transform Raman setup

**Fig. 1:**
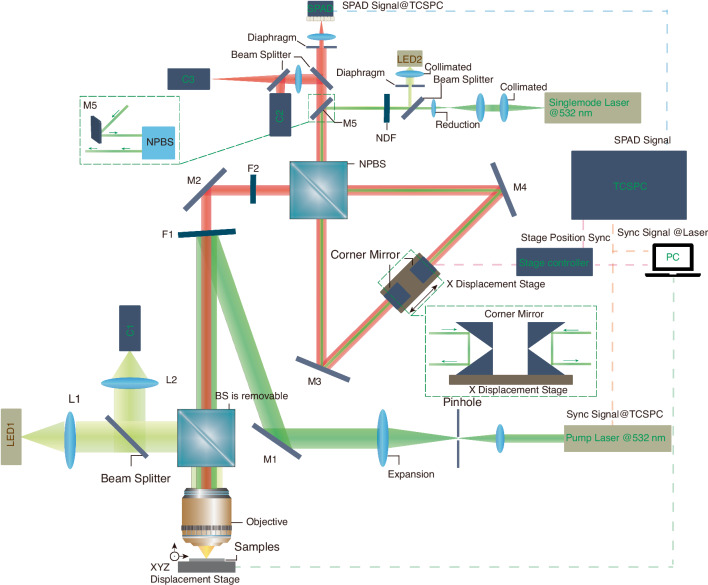
Sketch of time-resolved photon-counting Fourier-transform Raman setup. Elements are labeled (Pulsed laser: CNI, FL-532-PICO. Single-mode laser: CNI MSL-S-532. Filter F1, F2: Semrock BLP01-532R-25. NPBS: Union Optic, NPB0125-420-700, Corner mirrors: Thorlabs HR1015-P01. Stage: Aerotech ANT95L-075-E1-PL1-TAS. SPAD: Excelitas SPCM-AQRH-14. TCSPC: Picoquant, Multiharp 150 N. See the text for details)

The setup is shown in Fig. 1. A pulsed laser (532 nm, 100 ps pulse duration and 20 MHz repetition rate, linewidth below 5 cm^−1^) is used to excite the time-resolved Raman signal. It is mode filtered and expanded and reflected into the objective lens (100x/0.80 NA) by the filter F1 and focused onto the sample. An LED illumination and a camera coupled in via a removable beam splitter BS are used for reflection imaging of the sample.

The generated signal is collimated by the objective and enters the interferometer after passing through filter F1 and a filter F2 of the same type increasing the laser line suppression to more than 12 orders of magnitude. The interferometer design uses a non-polarizing beam splitter NPBS and adopts a vertically stacked design using corner mirrors on a high accuracy linear motor stage reflecting and lowering the beam, recombining the beams at the NPBS, providing outputs that are detected by the SPAD. The design of double oppositely moving mirrors achieves a doubling of the optical path and symmetry between both beams, improving the system resolution. The synchronization signal of the pulsed laser and the count signal of the SPAD are connected to a single photon counter. A three-dimensional displacement stage was used for sample lateral positioning and focusing. After an upgrade the second output of the interferometer was detected by a second SPAD to improve photon collection.

The second input of the interferometer is used, via mirror M5, to provide a white light reference and a 532 nm single longitudinal mode continuous wave (CW) laser to align and calibrate the interferometer. For more details of the setup see Materials and Methods.

### Photon signal processing and storage

**Fig. 2:**
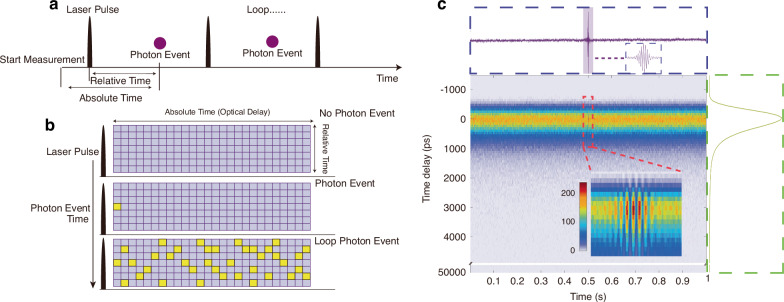
Recording photon data to obtain photon matrix. **a** Fine and coarse time of recording the photon event. **b** Data matrix of recorded photon events. **c** Measured data matrix values. Summing up the rows of the photon data matrix provides the interference matrix, while the sum of columns provides the time-resolved intensity. A locally enlarged view of the interference is provided (polystyrene (PS) sheet, *v*_*f*_ = 0.25 mm s^−1^, *T*_*f*_ = 1 s, *N*_*f*_ = 10)

We employ TCSPC to record the photon events into a photon data matrix (Fig. 2a) using a time-tagged time-resolved mode (T3 mode of the Multiharp 150 N). Each photon event is characterized by two timestamps: one fine, relative to the last laser pulse signal with 80 ps steps, and another coarse, relative to the start of the acquisition, counting the laser pulses received, having 50 ns steps. These two stamps are spanning the two dimensions of the data matrix, with the absolute time converted into an optical delay including corrections discussed below. When a photon event is detected, it is added to the photon count of the matrix element determined using the two time stamps (see Fig. 2b).

In an acquisition, the photon recording is started, then the stage is commanded to start a constant velocity motion between defined end points. After the acceleration phase, the constant velocity of the stage provides a constant time interval per optical delay change. The optical delay is determined from the coarse arrival time, and the photon is added into the corresponding element. For example, when testing plastic particles, we used a stage speed of *v*_*f*_ = 0.25 mm s^−1^ and a scan time of *T*_*f*_ = 1 s. We create a matrix of 120 × 10,000 elements covering a fine time range of (-1200 – 50,000) ps. The laser peak intensity is taken as zero relative delay as later shown in Fig. 5c. To reduce matrix size, only rows 1–80 are representing 80 ps intervals up to 5200 ps, while rows 81–120 are logarithmically sampled keeping the relative time interval constant^36^ (see supplement Fig. S3). The 10,000 columns cover an optical path difference of 0.25 mm × 4 = 1 mm with a column spacing of 1 mm/10,000 = 100 nm. After the scan, all photons are sorted into the data matrix (Fig. 2c). By summing the signal over the relative time, we obtain the interferogram curve. Similarly, the emission dynamics curve is obtained by summing the column values.

### Position recording and calibration

To convert the time delay curve into the spectrum, we find the exact zero delay *t*_0_ by fitting the central peak with a parabola. We perform a complex Fourier transform of the median subtracted data and phase-correct the resulting spectrum for the non-zero zero-delay position by multiplying with $\exp (i\omega {t}_{0})$, and then take the real part of the resulting spectrum for positive frequencies to determine the spectral intensity. This is equivalent to taking the cosine transform as known in Fourier spectroscopy but allows to shift the time zero with sub-sample resolution.

**Fig. 3:**
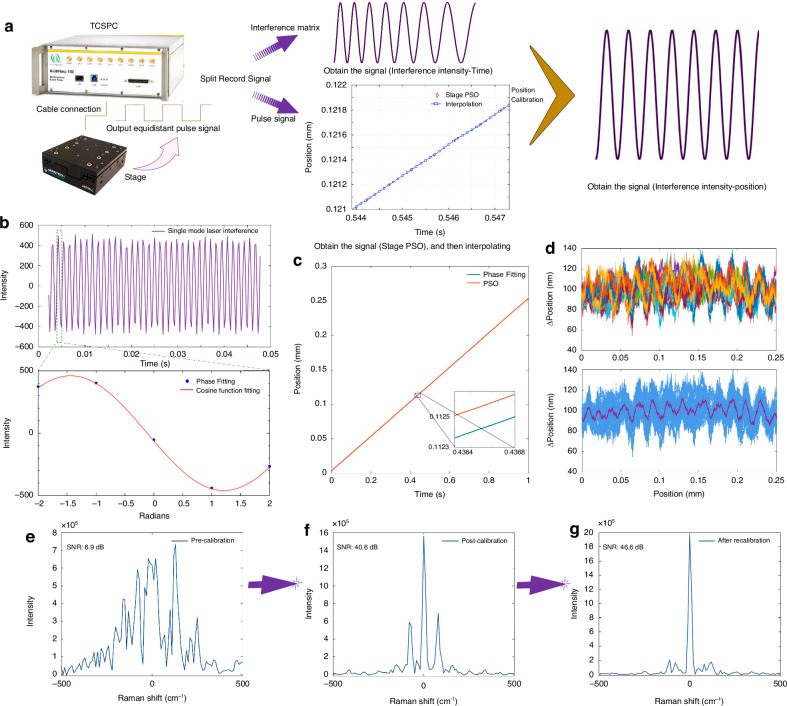
High-precision displacement stage data calibration. **a** Flowchart of interferogram position correction using stage PSO and interpolate the PSO 100 nm interval to 25 nm. **b** Single-mode laser interference intensity and cosine phase fit, using amplitude and phase as free parameters. **c** Stage position over time, determined from the phase fit (unwrapped), and the PSO output. **d** Position difference between the phase fit and the PSO output for 20 independent repetitions and its mean value. **e** Single-mode laser spectrum analyzed before position correction, SNR = 6.9 dB. **f** laser spectrum after PSO correction, SNR = 40.6 dB. **g** Laser spectrum after optical delay correction, SNR = 46.6 dB. Data taken at *v*_*f*_ = 0.25 mm s^−1^, *T*_*f*_ = 1 s, and *N*_*f*_ = 1, at about 6 MHz photon count rate using both APDs

The stage motion itself does not provide a sufficient accuracy in optical delay (which needs to be well below the wavelength of light), as can be seen in the resulting spectrum of a single-mode calibration laser shown in Fig. 3e (SNR = 6.9 dB), exhibiting a spectrum spread over a few hundred wavenumbers. To determine the optical delay with higher accuracy, we acquire the position of the stage in real time, by connecting the IO interfaces of the stage and the single-photon counter and use the stage’s position synchronized output (PSO) function. The PSO output is programmed to provide TTL pulses of 1 µs duration every 100 nm of stage sensor position increase (400 nm optical path length), which are recorded and time-tagged by the single-photon counter at the coarse time step resolution of 50 ns. We interpolate these measured positions linearly in time to obtain a position curve ${d}_{s}\left(t\right)$. We find that ${d}_{s}\left(t\right)$ deviates from the expected motion for constant speed with a residual of about ±50 nm, having both systematic and random parts across repeats, as shown in the Supplementary Fig. S4. Using $4{d}_{s}\left(t\right)/{c}$ as time delay in the Fourier transform, the spectral response is significantly improved, having a single central peak with two sidelines as shown in Fig. 3f (SNR = 40.6 dB).

To improve further, we determine the correct $d\left(t\right)$ from ${d}_{s}\left(t\right)$ using the interference signal measured for the single mode calibration laser. To do so we fit the interference phase at each point of the calibration laser interferogram over half of a period, as exemplified in Fig. 3b, resulting in a measured phase and thus optical delay as function of ${d}_{s}\left(t\right)$. The correct position $d\left(t\right)$ is then determined from the unwrapped phase $\rho \left(t\right)$ using

$$\,d\left(t\right)=\rho \left(t\right)\frac{\lambda }{8\pi }$$

where *λ* is the single mode laser wavelength. The phase fit and PSO output results have systematic and random variations. We measure the difference between the fit and PSO, $\Delta \left(t\right)={d\left(t\right)-d}_{s}\left(t\right)$ for 20 repeats and calculate its mean $\bar{\Delta }\left(t\right)$, as shown in Fig. 3d, to isolate the systematic deviations. We then use 4(${\bar{\Delta }\left(t\right)+d}_{s}\left(t\right))/c$ to sort the photons into the optical delay bins of the matrix. The resulting spectrum of the single mode laser is shown in Fig. 3g (SNR = 46.6 dB), having weaker sidelines than Fig. 3f, and a linewidth only limited by the scan range.

### Instrument temporal and spectral resolution

The instrument time resolution is a convolution of the laser temporal shape and the detector temporal response function. Assuming Gaussian shapes of full width at half maximum ${\tau }_{l}$ for the laser, ${\tau }_{d}$ for the detector, and ${\tau }_{s}$ for the TCSPC, the instrument time resolution is

$${\rm{\tau }}=\sqrt{{{\rm{\tau }}}_{\text{l}}^{2}+{{\rm{\tau }}}_{\text{d}}^{2}+{{\rm{\tau }}}_{\text{s}}^{2}}$$

**Fig. 4:**
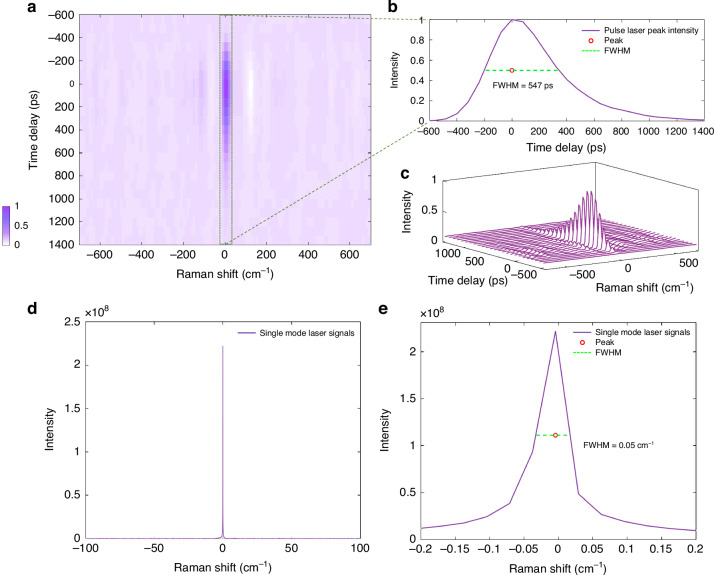
System time resolution and maximum spectral resolution results. **a** Emission measured from the pulsed excitation laser recorded using *P*_*l*_ = 2.5 mW, *v*_*f*_ = 0.25 mm s^−1^, *T*_*f*_ = 1 s, and *N*_*f*_ = 20, shown as 2D colormap limited to a time delay range of −600 to 800 ps and a spectral range of 300 to 4500 cm^−1^. Data taken with filter F2 removed. **b** Raman peak intensity profile with a FWHM of 547 ps. **c** Raman spectral time delay waterfall plot (time delay −600 to 1400 ps, spectral range −700 to 700 cm^−1^). **d** Single-mode las**e**r Raman spectrum. **e** Zoom around the laser peak showing a FWHM of 0.05 cm^−1^ limited by the scan range (Single mode laser, *v*_*f*_ = 0.25 mm s^−1^, *T*_*f*_ = 280 s, and *N*_*f*_ = 1.)

Using the nominal values for the laser ${\tau }_{l}$ = 100 ps, the TCSPC ${\tau }_{d}$ = 141 ps, and the SPAD *τ*_s_ = 350 ps, we find *τ* = 390 ps, dominated by the SPAD. The TCSPC provides 80 ps bin width, and each bin provides a spectrum as shown in Fig. 4. These data were taken for *P*_*l*_ = 2.5 mW laser power at the sample, a silver-coated mirror, using *v*_*f*_ = 0.25 mm s^−1^, and *T*_*f*_ = 1 s, and summed over *N*_*f*_ = 100 repetitions. The Fourier transform of 100 nm optical delay steps provides a spectral range of 0 to 50,000 cm^−1^, and we show here the spectral range around the excitation laser at 18,797 cm^−1^, with the Raman shift given by the difference of laser wavenumber minus the detection wavenumber.

We can see that the spectrum of the pulsed laser is well resolved (Fig. 4a, c). The time resolution of the instrument is 547 ps as measured in the Raman peak intensity (Fig. 4b), close to the estimated value of 390 ps from the hardware specifications.

We note that the 1 mm optical path length corresponds to a ± 0.5 mm optical path of the two interferometer arms, providing a delay of ±1.67 ps. This is well below the instrument time-resolution. At 100 mm optical path length, providing 0.1 cm^-1^ spectral resolution, the delay is ±167 ps, comparable to the time-resolution. The travel range of the stage is 70 mm, providing an optical path length of 280 mm, ±467 ps delay, and a nominal spectral resolution of 0.036 cm^−1^. We verified this using the calibration single mode laser as shown Fig. 4d, e. The observed spectral FWHM of 0.05 cm^−1^ is close to the nominal one. We also performed a multi-line resolution test on the Ne-Ar light source with 0.5 cm^−1^ nominal resolution (see supplement Fig. S5, Table S1), demonstrating that also when measuring >20 spectral lines all linewidths are below 1 cm^−1^, close to the nominal one.

### R6G-PVA-Si sample

**Fig. 5:**
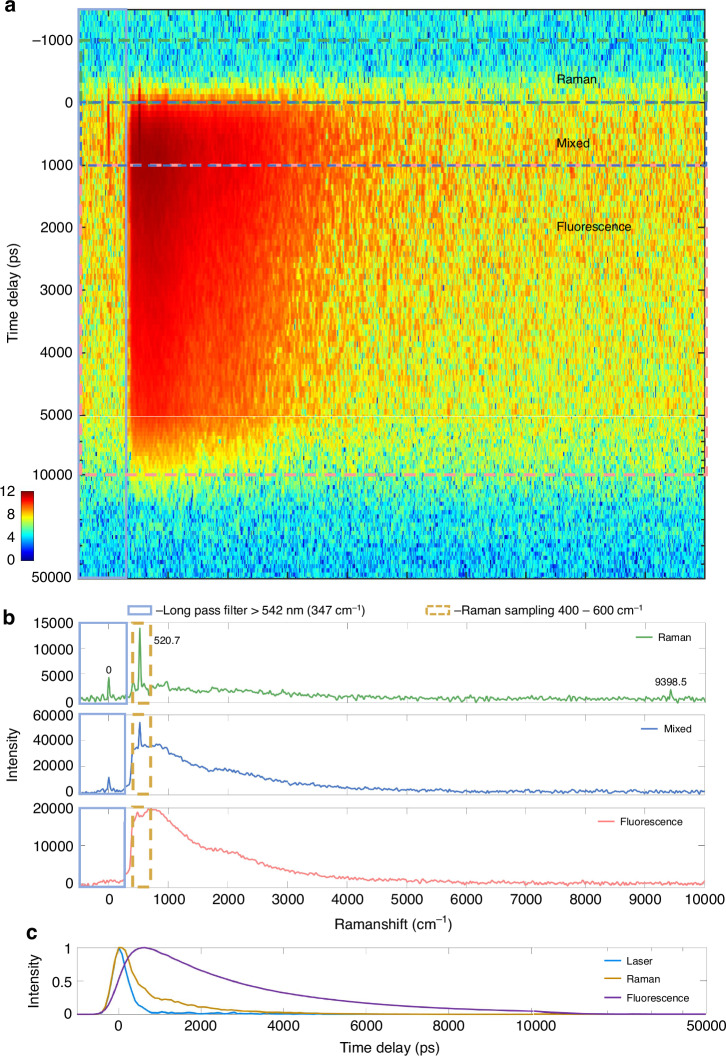
R6G-PVA-Si sample Raman fluorescence signal separation results image. **a** Emission 2D map (time-delay range −1200 to 50,000 ps, spectral range −500 to 10,000 cm^−1^). **b** Separation of Raman and fluorescence spectra at different time delays (Raman (−1000 to 0 ps), mixed (0 to 1000 ps), and fluorescence (1000 to 5000 ps)). **c** Temporal dynamics of laser (−20 to 20 cm^−1^), Raman (400 to 600 cm^−1^ of Si) and fluorescence (600 to 10,000 cm^−1^)

The R6G-PVA-Si sample was measured using *P*_*l*_ = 1 mW, *v*_*f*_ = 0.25 mm s^−1^, *T*_*f*_ = 1 s, and *N*_*f*_ = 100. The results are shown over a time delay range of −1500 to 50,000 ps, and a Raman spectral range of −1200 to 10,000 cm^−1^, creating the 2D map shown in Fig. 5a. We find that the emission contains significant fluorescent signals. The pulsed laser is at 0 cm^−1^. A peak at 9398.5 cm^−1^ is visible, which corresponds to a wavelength of 1064 nm, a residual of the pulsed laser before frequency doubling. The silicon Raman peak at 520.7 cm^−1^ is present around zero delay within the instrument time resolution. We can categorize the delay into three regions (Raman, mixed, and fluorescence), with the corresponding spectra shown in Fig. 5b, demonstrating significant separation of Raman and fluorescence. Additionally, we conducted comparative experiments on two mineral samples (Artificial Rainbow Carborundum and Amazonyte) using continuous-wave (CW) Raman systems with 532 nm and 785 nm excitation lasers, benchmarking against Fourier-transform time-gated Raman spectroscopy (see supplement Fig. S6). Comparing the dynamics of the silicon Raman peak and the fluorescence in Fig. 5c, it is apparent that the Raman signal occurs simultaneous with the laser excitation, while the fluorescence is rising over the instrument time resolution and later shows an approximately exponential decay with a lifetime of about 5 ns. Consequently, the time-gated Raman system developed in this study successfully separates the Raman and fluorescence signals, resulting in a purer Raman spectrum. FSC^3^ of 2D time-frequency spectra will lead to a purer separation between Raman and fluorescence, as we will report in a forthcoming work.

### PMMA-PS-mrobeads-R6G sample

**Fig. 6:**
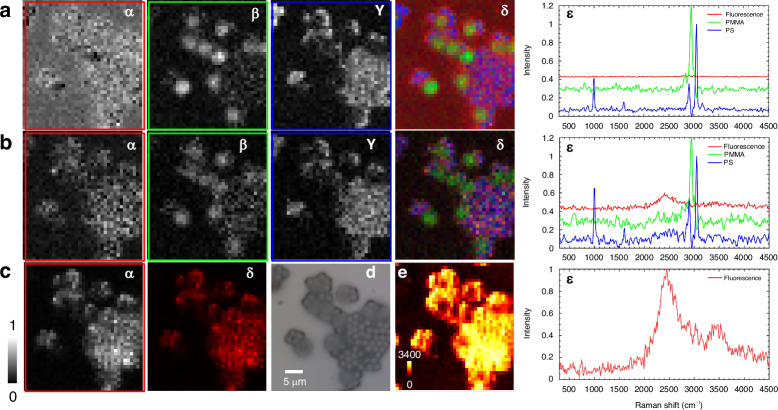
Imaging of a sample with 5 µm PMMA and 2 µm PS beads with R6G. Results of FSC^3^ analysis for three time-delay ranges. Rows are **a** Raman, **b** mixed, and **c** fluorescence time windows. Columns are component concentrations of (α) Fluorescence, (β) PMMA Raman (γ) PS Raman, (δ) Pseudo-color image of α (red), β (green), and γ (blue). (ε) component spectra for the different time ranges. **d** Reflection image. **e** Fluorescence lifetime analysis (for details see supplementary Fig. S8), color scale as shown in picoseconds

To demonstrate the system for imaging, two types of spheres (PMMA and PS) were mixed with R6G such that both spheres were accompanied by a fluorescent background signal, see Materials and Methods. Data were taken using *P*_*l*_ = 1 mW, *v*_*f*_ = 0.25 mm s^−1^, *T*_*f*_ = 1 s, *N*_*f*_ = 1, and planar scanning across (37 × 40) µm^2^ with 1 µm steps. The resulting spectra were noise-reduced using SVD (see Supplementary Fig. S7), separately for the time-windows of Raman, mixed, and fluorescence, and then analyzed using FSC^3^ for component extraction, resulting in the data shown in Fig. 6.

For the Raman time window in Fig. 6a, the characteristic Raman spectra of the two spheres are found, and the spatial distributions of the corresponding concentrations in α and β. The 5 µm beads are larger than the focal depth and their center is away from the focus, as seen in the white-light reflection image Fig. 6d. This results in a reduced Raman signal. In the mixed range (Fig. 6b), the fluorescence intensity dominates, but the two types of spheres remain distinguishable. In the fluorescence range (Fig. 6c), the Raman signal is absent, and the two types of spheres are not distinguished. Only a single component is retrieved. These results demonstrate that the time-resolved photon counting Fourier-transform Raman system developed in this study can effectively distinguish between Raman and fluorescence signals, yielding a pure Raman signal.

## Discussion

To address the limitations of spectral range and spectral resolution inherent to current grating-type Raman systems using array detectors, and allow for separation and simultaneous detection of Raman and fluorescence by their temporal dynamics, we have devised a time-resolved photon-counting Fourier-transform Raman platform. The platform exhibits high operational stability and a low detection limit (see supplement Figs. S9, S10). Notably, using a high-quality linear stage, position marker recording and position correction via a calibration laser, we can assign optical delay to the absolute arrival time of photons measured in 50 ns time bins, at the same time as recording their relative timing to a pulsed laser source to measure the emission dynamics with 80 ps time bins, recorded in a time tagged time-resolved mode. This allows a fast continuously scanning stage, enabling the acquisition of a 2D spectrum per second (the scanning speed can be increased by shortening the total optical range difference, see Supplementary Fig. S11). We show a time resolution of 547 ps (FWHM) and adjustable spectral resolution to accommodate diverse sample requirements. The system features an extended spectral coverage from −1000 to 10,000 cm⁻¹ and a maximum spectral resolution of 0.05 cm⁻¹ (FWHM) thereby meeting the objectives of high temporal resolution, wide spectral range, and high spectral resolution. The wavelength range is limited only by the spectral properties of the optical components used. For the present setup the range is determined by the spectral filter used (>542 nm) and the SPAD detector quantum efficiency (for 520 to 860 nm more than 50%, for 410 to 1010 nm more than 10%), which allows to detect from 542 nm to about 950 nm within a factor of 3 of sensitivity, covering a wavenumber range of about 8000 cm^−1^.

The separation of Raman and fluorescence signals was verified using silicon with R6G with prominent Raman peaks, establishing that a relatively pure Raman signal could be obtained within the time delay range of −1000 to 0 ps. To demonstrate the imaging capability of the instrument we used plastic microspheres exhibiting fluorescence, and showed that two plastic types and fluorescence can be separated using FSC^3^ analysis. This analysis could be improved by analyzing spectro-temporal components, as introduced in ref.^37^, which should allow to separate Raman and fluorescence components with high fidelity.

Importantly, while the high spectral resolution shown is not required for Raman spectra of condensed matter, for gas-phase spectroscopy, linewidths are typically given by Doppler broadening around 0.01 to 0.1 cm⁻¹, making full use of the high resolution offered by the instrument. In this case 1 ns pulses of Fourier-limited spectral width of 0.02 cm⁻¹ could be used, allowing to retain the spectral resolution while no being limited by the time-resolution of the SPAD detectors used.

Notably, the concept shown here could be extended to other applications including quantum technology to measure spectrally and time-resolved photon correlations. Significant improvements of instrument performance are feasible. An improvement of the time-resolution to 10 ps is possible using superconducting nanowire single photon detectors (SNSPD). A corresponding excitation pulse of 10 ps duration has a transform-limited linewidth around 2 cm^−1^ and is thus still suitable for high resolution Raman imaging. Furthermore, delay scanning could be accelerated using voice-coil actuators to allow for 100 scans per second. Polarization resolution can be implemented, relevant both for Raman imaging and quantum optical applications. Both SNSPD and broadband SPADs can extend the spectral detection range of the instrument to the near-infrared (>950 nm) or ultraviolet (<400 nm), enabling broader scientific applications.

## Materials and methods

### R6G – PVA - Si sample

Polyvinyl alcohol (PVA, MACLIN) particles (1 g) were placed in a beaker with 20 g of water, which was placed on a heating plate for 25 min at a temperature of 75 °C, with a stirrer (10 mm × 30 mm) at 550 rpm, to completely dissolve the PVA. 10 μL of 5 × 10^−6 ^mM Rhodamine 6 G (R6G, Sigma-Aldrich) was added. Once the R6G was fully mixed, 10 μL of the solution was drop-cast onto a Si crystal (10 mm × 10 mm size, thickness 0.5 mm) and allowed to dry.

### PMMA - PS microbeads - R6G sample

We used standard-sized microspheres (YUAN Biotech, China). 50 μL of both 2 μm polystyrene (PS) and 5 μm polymethyl methacrylate (PMMA) beads, with a concentration of 25 mg/mL, was mixed into 10 mL of water. Then, 10 μL of 10^−9 ^mM R6G solution was added. 10 μL of the solution was drop-cast onto aluminized glass substrates (aluminium film thickness 25 nm, 75 mm × 25 mm size 1mm thickness) and dried.

### Time-resolved photon-counting Fourier-transform Raman setup details

Two lasers are used. A pulsed laser (CNI, FL-532-PICO, 50 mW CW power, 532 nm center wavelength, 100 ps pulse duration and 20 MHz repetition rate, linewidth below 5 cm^−1^) is used to generate the pulsed Raman signal. The pulsed laser is mode filtered and expanded (*f* = 20 mm, Thorlabs, AC080-020-A, and *f* = 100 mm, Thorlabs, AC254-100-A, pinhole (50 μm, Thorlabs, P50HCB)) and reflected into the objective lens (Olympus LMPlanFLN 100x/0.80 NA) of 2.9 mm back-focal size, via a mirror M1 and the filter F1 (Semrock BLP01-532R-25) and then focused by the objective onto the sample. LED1 (Thorlabs MWWHL4), together with a camera C1 (Do3think, U3P2100-H), lenses L1, L2 (Thorlabs, AC254-125-A-ML), and 50:50 beam-splitters (Thorlabs, BSW10R) are used for reflection imaging of the sample.

The generated Raman signal is collimated by the objective and enters the interferometer after passing through filter F1 (Semrock BLP01-532R-25) and a filter F2 of the same type increasing the laser line suppression to more than 12 orders of magnitude. The interferometer design uses a non-polarizing beam splitter NPBS (Union Optic, NPB0125-420-700, 45:45 ± 5%, 1 inch size) and adopts a vertically stacked design using corner mirrors (Thorlabs HR1015-P01) on a high accuracy linear motor stage (Aerotech ANT95L-075-E1-PL1-TAS) reflecting the beam and lowering the beam by 1 cm, recombining the beams at the NPBS, providing outputs that are detected by the SPADs (SPAD, Excelitas SPCM-AQRH-14, focusing lens, f = 19 mm (Newport, KPX040AR.14), and an adjustable diaphragm set to 2.9 mm diameter (Thorlabs SM05D5D). The SPAD was chosen for its high quantum efficiency of 55–70% (540–830 nm) and high maximum count rate of 30 MHz. The design of double oppositely moving mirrors achieves a doubling of the optical path and symmetry between both beams, improving the system resolution. The synchronization signal of the pulsed laser and the count signal of the SPAD are connected to a single photon counter (Picoquant, Multiharp 150 N). A three-dimensional displacement stage (Jancheng Optoelectronics XY2-2550, VS-0805) with a range of 25 mm in XY and 5 mm in Z, with a resolution of 50 nm, was used for sample lateral positioning and focusing. After an upgrade the other output of the interferometer was detected by a second SPAD to improve photon collection (see Supplementary Figs. S1, S2).

The second input of the interferometer is used, via mirror M5, to provide two sources. Firstly, a white light reference consisting of LED2 (Thorlabs MWWHL4), a collimating lens *f* = 50 mm, (Thorlabs LA1131-A), a diaphragm set to 1 mm diameter (Thorlabs SM05D5D), to find zero delay. Secondly, a 532 nm single longitudinal mode continuous wave (cw) laser (CNI MSL-S-532) coupled via a single-mode polarization-maintaining optical fiber (Thorlabs, P3-488PM-FC-2) of 50 mW maximum power with a coherence length of 50 m to align and calibrate the interferometer. After collimating the fiber output with an *f* = 8 mm lens (Thorlabs, C240TMD-A), the beam diameter is 1 mm. The beam is attenuated by a 0.1% neutral density filter NDF (Thorlabs NE30A) after coupling it into the beam path via a 50% beam splitter (Thorlabs BSW10).

### Data analysis

The data acquired using the Raman spectroscopy system were analyzed using FSC^3^ ^37–39^, an analysis tool which decomposes hyperspectral images into a linear combination of chemical components defined by their non-negative Raman spectra and spatial v/v concentration distributions. This method was previously applied to a range of hyperspectral data including coherent Raman scattering^37^, spontaneous Raman^39^, and IV-LEEM^40^. Here, the spectra of the plastic beads are determined as spatial average in regions corresponding to beads of different chemical composition and used as fixed components in the factorization algorithm, which determines their spatial distribution and the spectrum and concentration of an extra component, which can be identified as the surrounding matrix. The component spectra are normalized so that their spatial concentration maps have unity maximum value.

## Supplementary information

Supplementary Information

## Data Availability

All data needed to evaluate the conclusions in the paper are present in the paper. Additional data related to this paper may be requested from the authors.
