# Supplementary material for: Time-resolved photon counting Fourier-transform micro-spectroscopy enables simultaneous Raman and fluorescence lifetime imaging
Source: Light Sci Appl. 2025 Oct 29;14:378. doi: 10.1038/s41377-025-02020-8 (PMC12569202; doi:10.1038/s41377-025-02020-8)
Supplement: Supplementary file 1 — Supplementary Information [file 41377_2025_2020_MOESM1_ESM.docx]

**Supplementary Information for**

**Time-resolved photon counting Fourier-transform micro-spectroscopy enables simultaneous Raman and fluorescence lifetime imaging**

**Lindong Shang^a,b,c^, Xiaodong Bao^a,b,c^, Hao Peng^a,b,c^, Fuyuan Chen^a,b,c^, Yu Wang^a,b,c^, Kunxiang Liu^d^, Peng Liang ^a,b,c,e^, Yuntong Wang^a,b,c^,** **Xusheng Tang^a,b,c^, Francesco Masia^f^, Wolfgang Langbein^g^*, Bei Li ^a,b,c,e^***

*a. Changchun Institute of Optics, Fine Mechanics and Physics, Chinese Academy of Sciences, Changchun, 130033, China*

*b. University of Chinese Academy of Sciences, Beijing, 100049, China*

*c. State Key Laboratory of Advanced Manufacturing for Optical Systems, Chinese Academy of Sciences, Changchun, 130033, China*

*d. Shenzhen Institute of Advanced Research, University of Electronic Science and Technology of China, Shenzhen 518038, China*

*e. Hooke Instruments, Changchun, 130033, China*

*f. School of Biosciences, Cardiff University, Cardiff, CF10 3AX, UK*

*g. School of Physics and Astronomy, Cardiff University, Cardiff, CF24 3AA, UK*

**Correspondence:* [*langbeinww@cardiff.ac.uk*](mailto:langbeinww@cardiff.ac.uk)*;* [*beili@ciomp.ac.cn*](mailto:beili@ciomp.ac.cn)

| Contents | Description |
| --- | --- |
| Supplementary Fig. 1 | Double detector optical setup diagram. |
| Supplementary Fig. 2 | Double detectors increase photon collection efficiency. |
| Supplementary Fig. 3 | Photon matrix x-axis sampling distribution. |
| Supplementary Fig. 4 | Interferometer stage linearity and repeatability. |
| Supplementary Fig. 5 | Ne-Ar Source spectra peak position. |
| Supplementary Fig. 6 | Mineral Sample Scanning Test. |
| Supplementary Fig. 7 | Spectral SVD noise reduction. |
| Supplementary Fig. 8 | Fitting of fluorescence lifetime. |
| Supplementary Fig. 9 | Instrument stability analysis. |
| Supplementary Fig. 10 | Instrument minimum detection limit scan test. |
| Supplementary Fig. 11 | Shorter total optical path for faster scanning. |
| Supplementary Table 1 | Ne-Ar Source peak position and FWHM. |


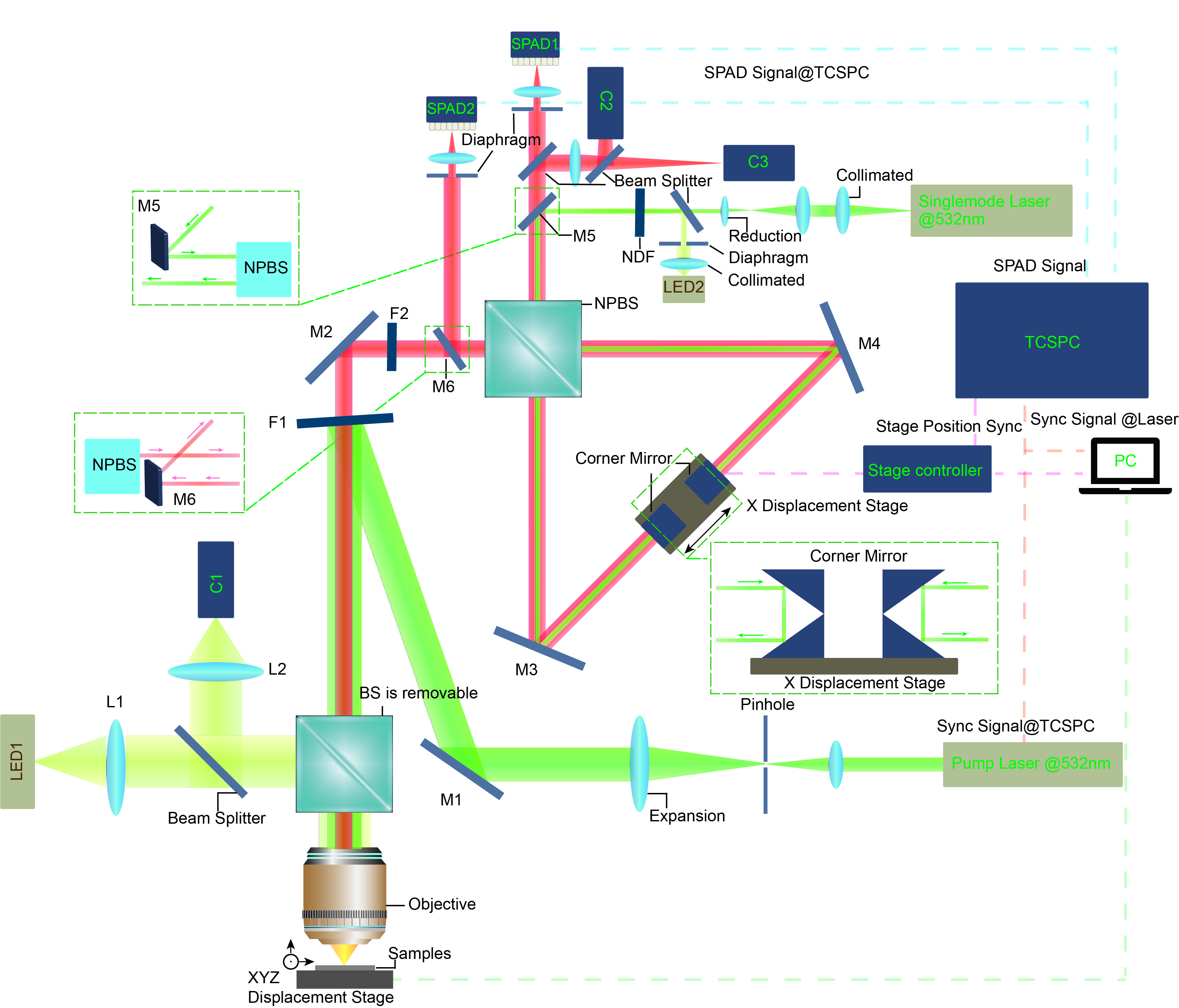


**Fig. S1** Double detector optical path setup diagram.

Fig. S1 shows a diagram of the optical setup after adding the second SPAD. It takes the light which exits the beamsplitter NPBS on the second port and reflects it towards the detector. The setup in front of the second detector is identical to the first one, with an aperture diaphragm (Thorlabs, SM05D5D) and a focusing lens (f=19 mm, Newport, KPX040AR.14). The detector signal line is connected to the second input channel of the TCSPC.


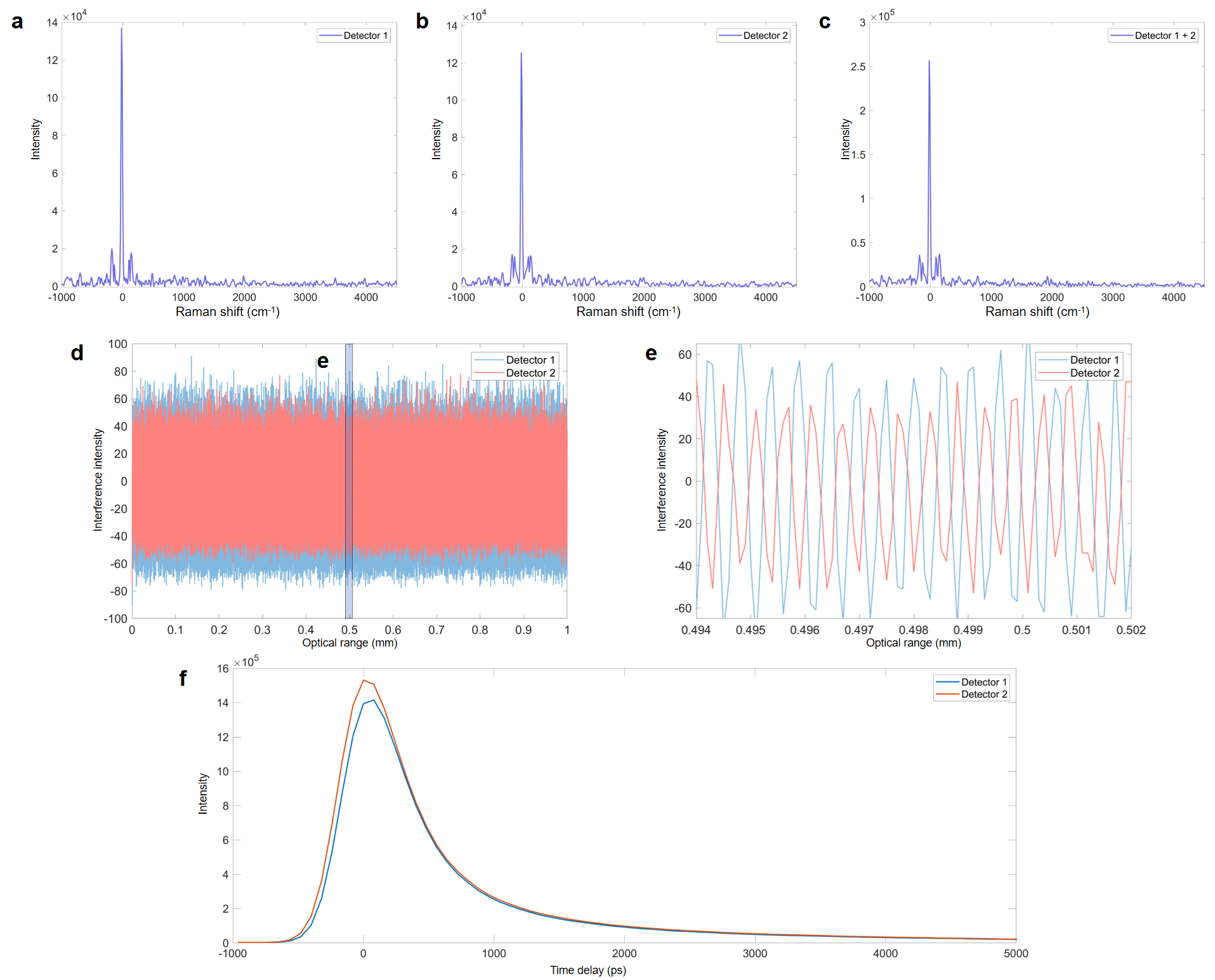


**Fig. S2** Double detectors increase photon collection efficiency. **a**, **b** shows the calibration laser spectra obtained using only the original **a** or only the additional **b** detector, and **c** shows the spectrum using the difference between the detectors. **d** interferograms of the two detectors for the calibration laser, with **e** showing a temporal zoom. Data taken with the calibration laser, $v_{f}$= 0.25 mm s^-1^, $T_{f}$= 1 s, and $N_{f}$= 1. **f** emission intensity dynamics recorded by the two detectors during the detection of PS. Data taken on a PS sheet, $v_{f}$= 0.25 mm s^-1^, $T_{f}$= 1 s, $N_{f}$= 100, pulsed laser power 2.5 mW.

Fig. S2 **a-c** show the calibration laser spectra obtained using the original **a** or the additional **b** detector, and using the difference between both detectors **c**. It can be seen that the spectral intensity is significantly enhanced after superposition, and the signal-to-noise ratio is higher. **d** shows the interferograms of the two detectors when detecting a single-mode laser, and **e** is a temporal zoom showing that the intensity oscillations seen on the two detectors are out of phase. To ensure accurate superposition, a suitable time delay is introduced in one of the interferograms during superposition to take into account the different time delay of the SPADs and the propagation time through the cables.

The emission intensity dynamics recorded by the two detectors during the detection of PS is shown in Fig. S2 **f**. The two curves are overlapped after considering a fixed propagation delay difference between the detectors. The two detectors are then synchronized.


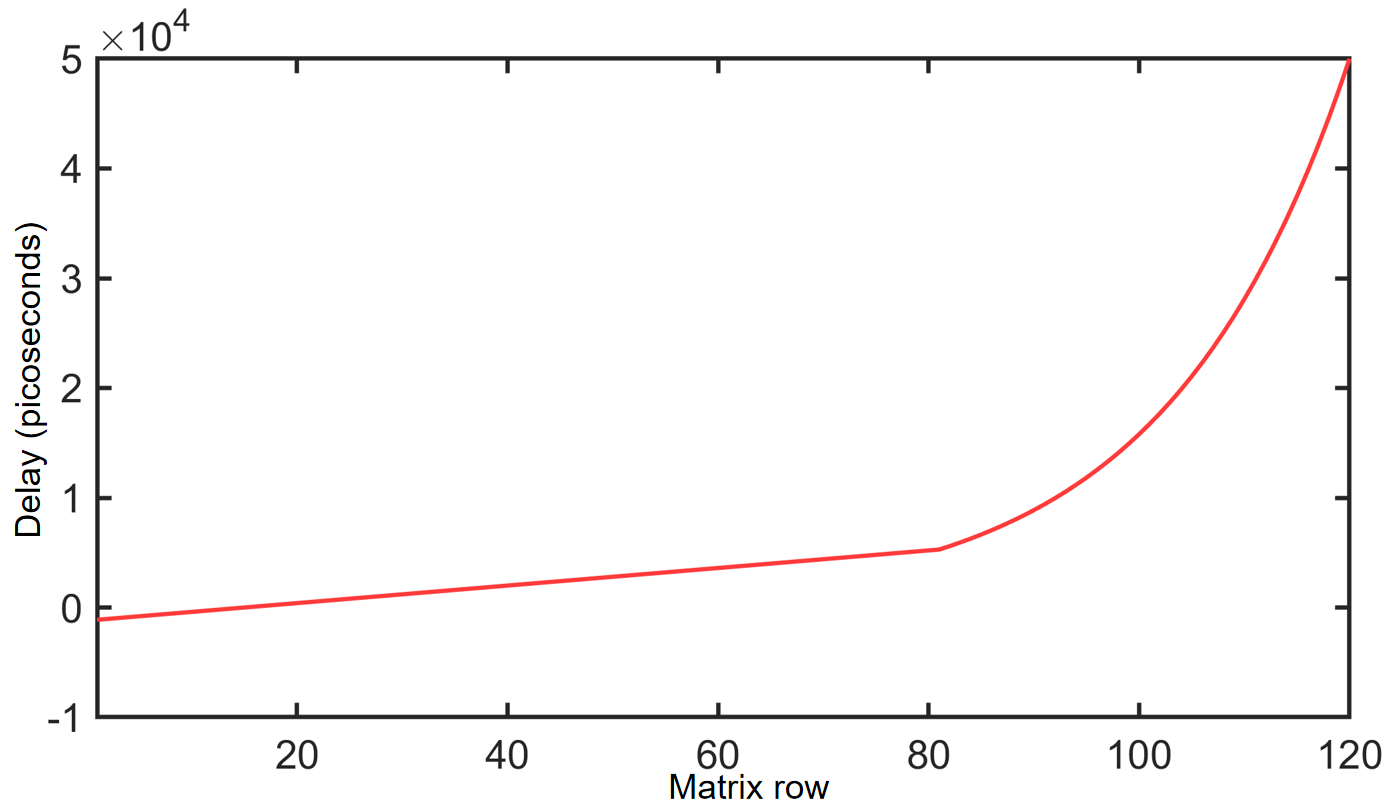


**Fig. S3** Photon matrix x-axis sampling distribution.

Fig. S3 shows the photon matrix x-axis sampling distribution. Since the vast majority of samples have fluorescence lifetimes below 5 ns, we used uniformly spaced linear sampling for the first 80 rows of data covering the time range -1200 to 5200 ps. In order to reduce the number of pixels, we use a logarithmic sampling method for the last 40 rows of data. This strategy can be changed according to actual needs.


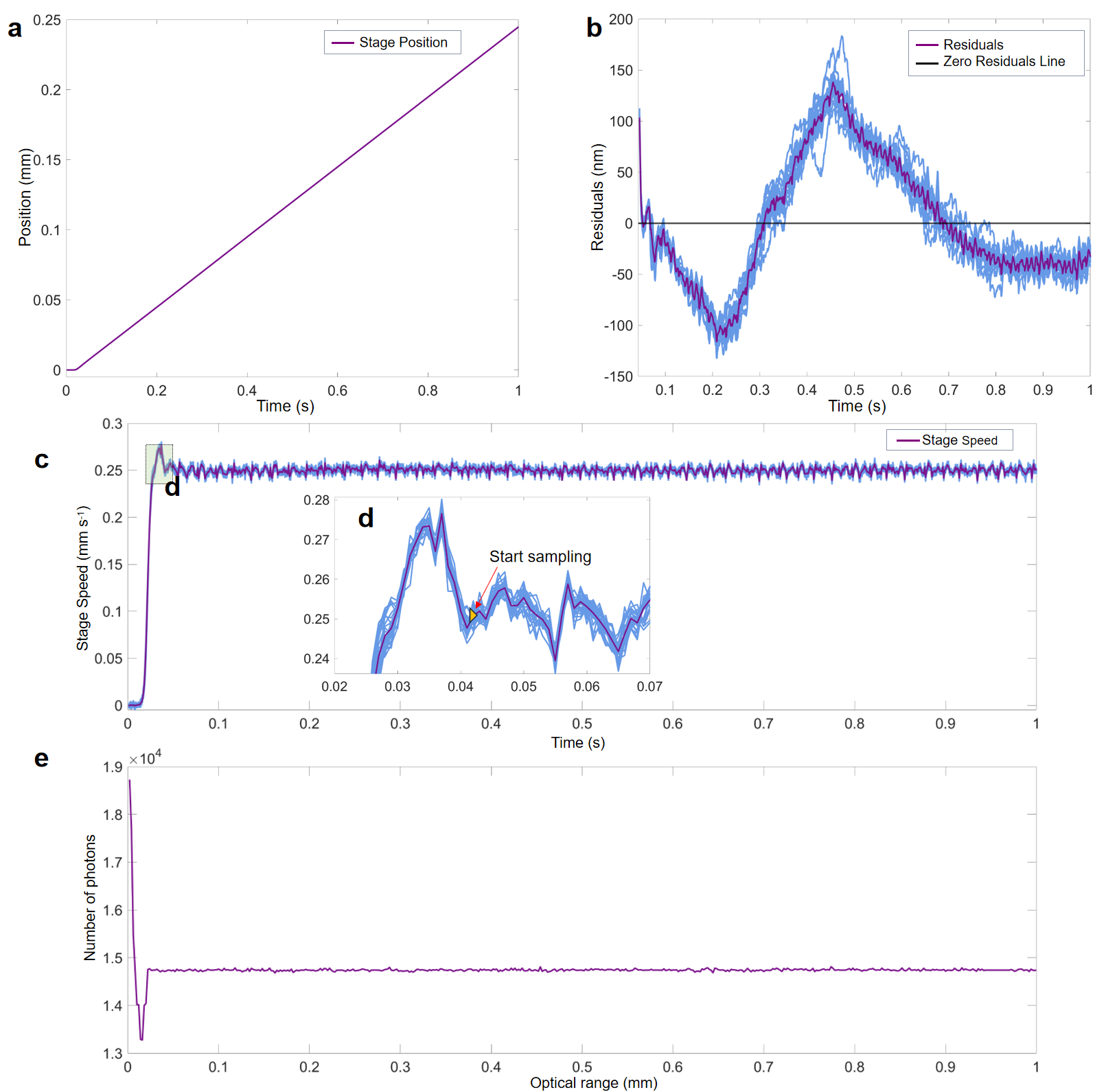


**Fig. S4** Detailed explanation of stage calibration. **a** Recorded PSO position over time $d_{s}\left( t \right)$ for a move with $v_{f}$= 0.25 mm s^-1^ and $T_{f}$= 1 s. **b** residuals of a linear fit to $d_{s}\left( t \right)$ for 20 repeated moves (blue thin lines), and their mean (red thick line). **c** Stage speed calculated from $d_{s}\left( t \right)$ for 20 repeated moves. **d** indicating the time at which the interferogram is started. **e** Number of photons detected per 2 µm position interval, using only one arm of the interferometer to avoid modulation by interference. Data taken with the calibration laser, $v_{f}$= 0.25 mm s^-1^, and $T_{f}$= 1s, summed over $N_{f}$= 1 repetition, photon rate about 7.4 MHz.

Further details of the analysis of stage motion: Fig. S4 **a**, **b** show that the stage position is not perfectly linear with time. There are -150 to 150 nm deviations from the linear motion commanded for the stage. The motion $d_{s}\left( t \right)$ is recorded with the PSO function. A linear fit is done over the time range from 0.042 to 1 s and the residuals are shown in **b** for 20 repetitions of the same linear motion. The residuals show a dominant systematic component, with a random component on the ±20 nm scale. **c** shows measured speed over 1ms intervals during the test. **e** shows the number of photons per 2 µm interval over a single scan during the test. The stage speed $v_{f}$is accelerated from 0, which results in the ideal position and speed of motion not being achieved at the beginning of the motion. During the stage acceleration phase the velocity and photon number change significantly, thus we avoid this phase. We choose a point **d** after the end of the acceleration phase to get its timestamp (0.042 s). From **a**, we can get its position point is 0.005 mm, and we collect the interferogram from that point. We acquire the data between 0.005 - 0.255 mm of stage movement (total optical path difference multiplied by 4 is still 1mm, and sampling time is still 1s).


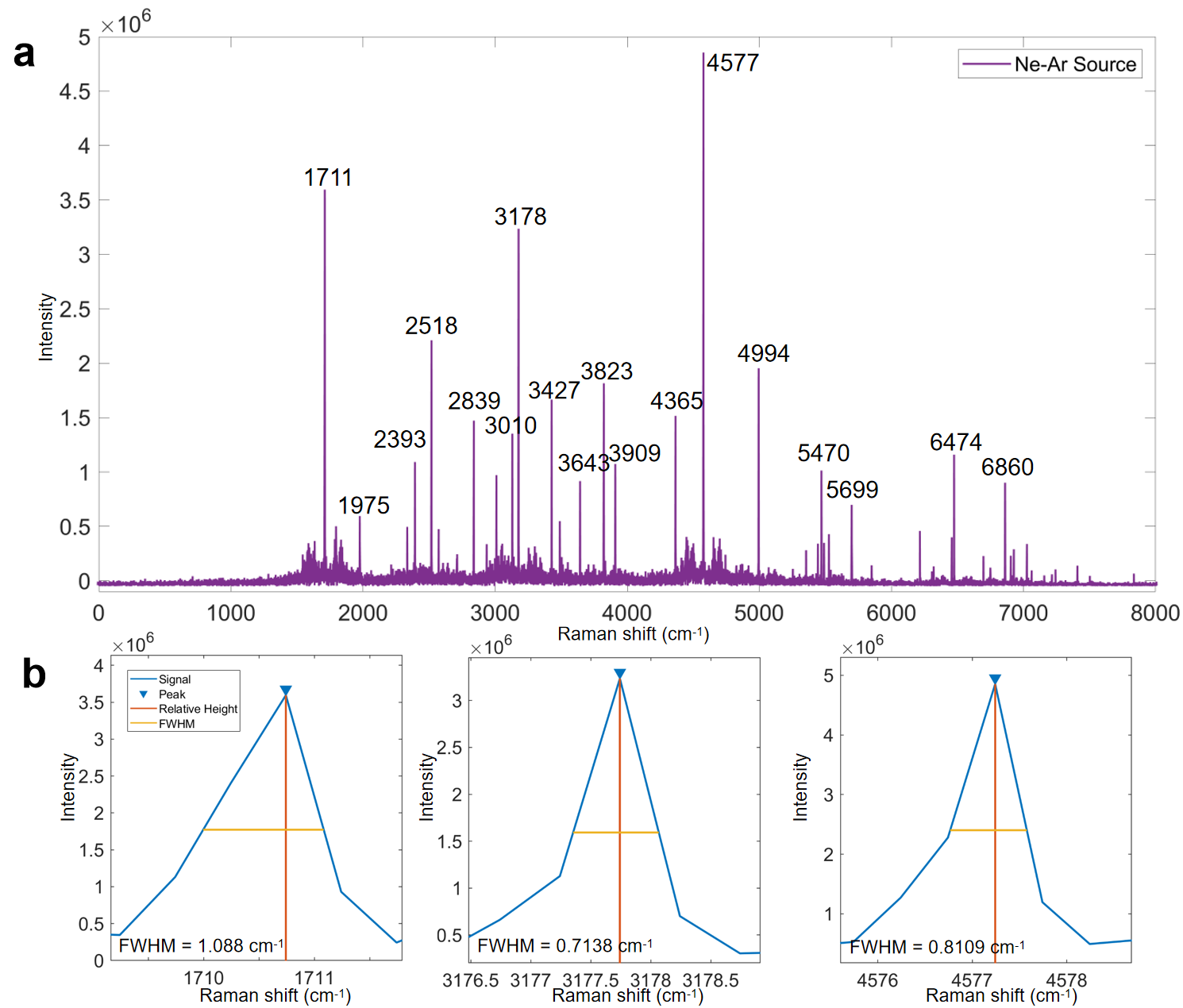


**Fig. S5** The Ne-Ar Source peak position spectra and the full width at half maximum (FWHM) of the main peaks. **a** Ne-Ar Source spectrum. **b** FWHM of the peaks at 1711 cm⁻¹, 3178 cm⁻¹, and 4577 cm⁻¹. $v_{f}$= 0.25 mm s^-1^,$T_{f}$= 20 s, $N_{f}$=1, spectral resolution 0.5 cm^-1^. Shown is the spectral range 0 to 8000  cm^-1^.

Fig. S5 displays a higher spectral resolution spectrum of a Ne-Ar source. Multiple peaks are observed within the 0 to 8000 cm⁻¹ range, and we have statistically analyzed the full width at half maximum (FWHM) of the major peaks (​​Table S1​​). Most peaks exhibit FWHM values between 0.5 and 1.1 cm⁻¹, while the strongest peaks at ​​1711 cm⁻¹​​, ​​3178 cm⁻¹​​, and ​​4577 cm⁻¹​​ show FWHM values of ​​1.09 cm⁻¹​​, ​​0.714 cm⁻¹​​, and ​​0.811 cm⁻¹​​, respectively, indicating high spectral resolution. Additionally, these peaks demonstrate excellent signal quality and high signal-to-noise ratios (SNR).

**Table S1.** Raman shifts and full widths at half maximum (FWHM) of the major Ne-Ar Source peaks.

| Raman Shift (cm^-1^) | Wavelength (nm) | Peak position FWHM (cm^-1^) |
| --- | --- | --- |
| 1711 | 585.3 | 1.09 |
| 1975 | 594.5 | 1.04 |
| 2393 | 609.6 | 1.05 |
| 2518 | 614.3 | 0.59 |
| 2839 | 626.7 | 0.54 |
| 3010 | 633.4 | 0.74 |
| 3178 | 640.3 | 0.71 |
| 3427 | 650.6 | 0.87 |
| 3643 | 659.9 | 0.64 |
| 3823 | 667.8 | 0.72 |
| 3909 | 671.7 | 1.08 |
| 4365 | 692.9 | 0.65 |
| 4577 | 703.2 | 0.81 |
| 4994 | 724.5 | 0.76 |
| 5470 | 750.4 | 0.73 |
| 5699 | 763.5 | 0.89 |
| 6474 | 811.5 | 0.65 |
| 6860 | 837.7 | 0.77 |


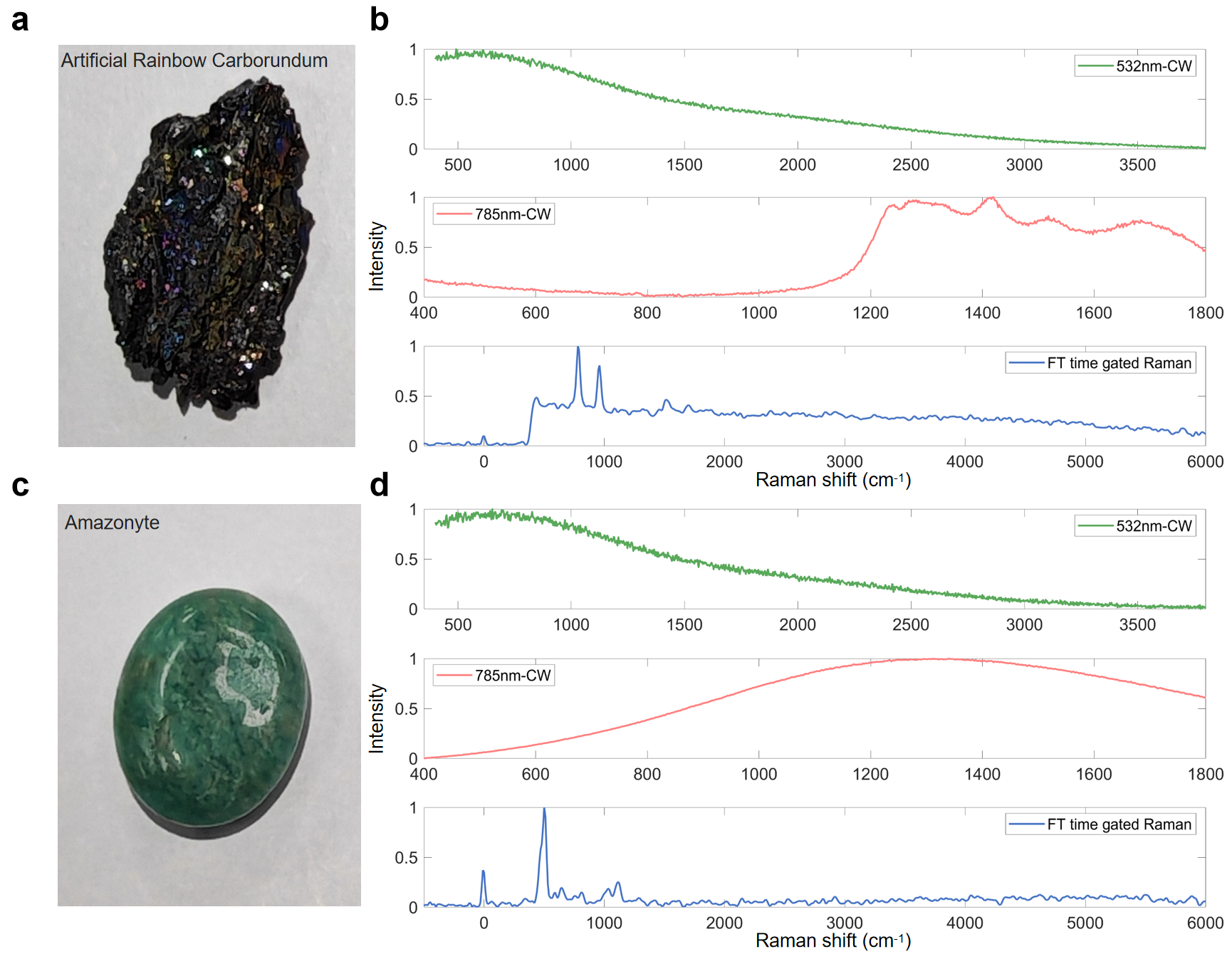


**Fig. S6** Mineral sample results. **a** Picture of artificial Rainbow Carborundum sample and **b** corresponding Raman spectra. ​​532 nm continuous-wave (CW) laser​​, (Hooke P300, CW-laser power 10 mW, exposure time 5s, 350 to 3800 cm^-1^). ​​785 nm CW laser​​, (Hooke D100, CW-laser power 20 mW, exposure time 5 s,400 to 1800 cm^-1^). 532 nm Fourier-transform time gated Raman, ($v_{f}$= 0.25 mm s^-1^, $T_{f}$= 1 s, $N_{f}$= 100, pulsed laser power 5 mW, time delay -1000 to 0 ps). **c** Picture of Amazonyte sample and **d** corresponding Raman spectra. ​​532 nm continuous-wave (CW) laser​​, (Hooke P300, CW-laser power 15 mW, exposure time 5 s, 350 to 3800 cm-1). ​​785 nm CW laser​​, (Hooke D100, CW-laser power 20 mW, exposure time 5 s, 400 to 1800 cm^-1^). 532nm Fourier-transform time gated Raman, ($v_{f}$= 0.25 mm s^-1^, $T_{f}$= 1 s, $N_{f}$= 100, pulsed laser power 5 mW, time delay -1000 to 0 ps).

​​Fig. S6​​ demonstrates distinct Raman detection results for the two minerals. It can be observed that for both minerals, neither ​a 532 nm CW laser​​ nor a ​​785 nm CW laser​​ excitation yields discernible Raman peaks. However, when employing ​​our Fourier-transform time-gated Raman instrument​​ with a ​​delay setting of -1000 to 0 ps​​, Raman peaks are observed, demonstrating superior ​​fluorescence suppression​​. Furthermore​​, the time-gated Raman results of artificial Rainbow Carborundum reveal that the ​​fluorescence background​​ has not been completely suppressed. In our future work, ​​employing SPAD detectors with higher temporal resolution​​ is expected to further suppress fluorescence, yielding ​​cleaner Raman peaks.


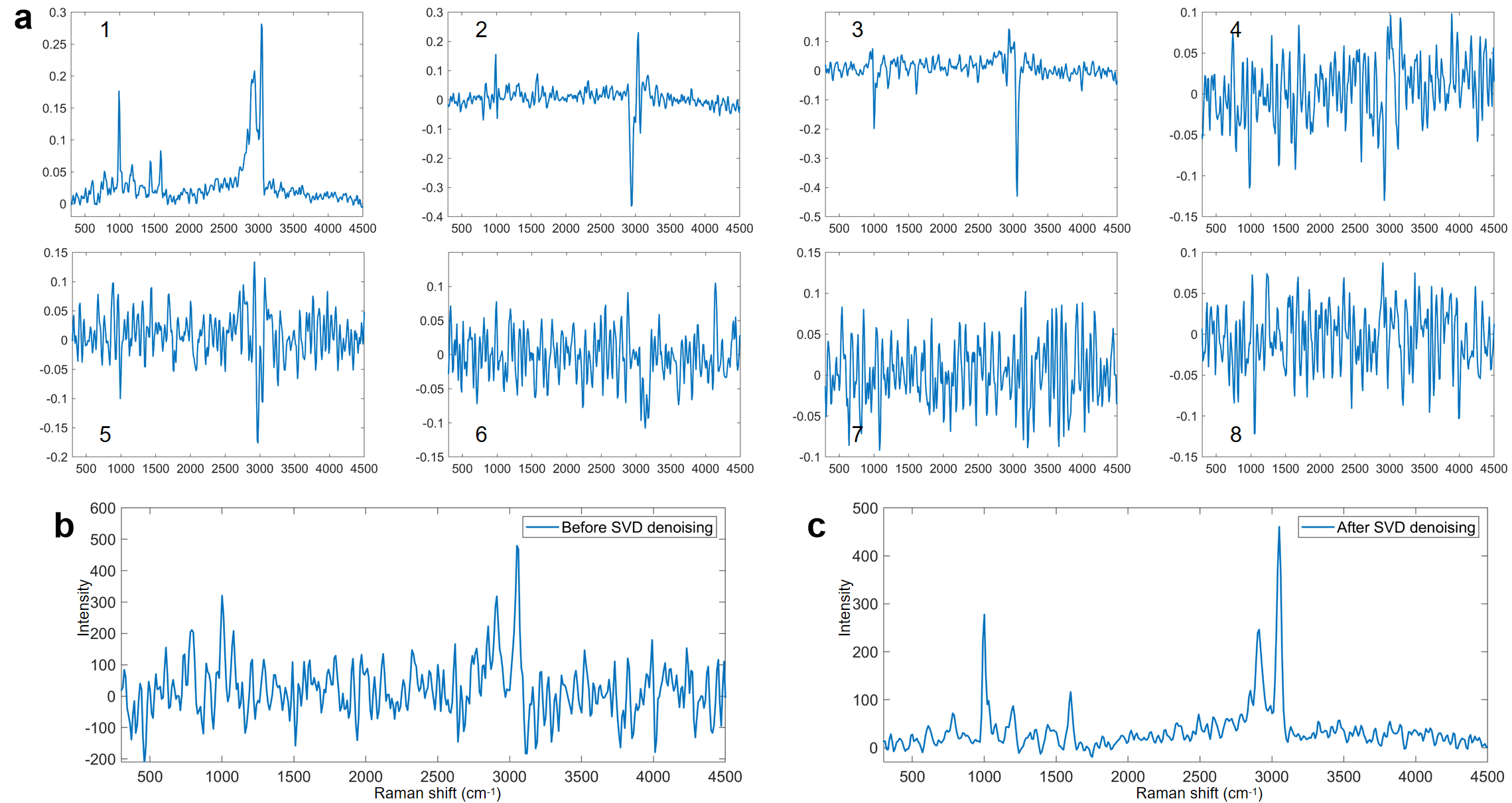


**Fig. S7** Example SVD denoising of Raman data (time range -1000 to 0 ps) over about 1500 spatial points, as shown in Fig. 6 of the main paper. **a** Spectra of the first 8 singular vectors of the SVD. **b** example spectrum of one position before SVD noise reduction. **c** spectrum of the same position after SVD noise reduction using 6 components.

Fig. S7 illustrates the SVD noise reduction process of the spectra using in the imaging shown in Fig.6 of the main paper. **a**, the 8 significant SVD components and the top 6 most important SVD spectra that are retained. **b**,**c** Raman spectrum at one position, before **b** and after **c** SVD noise reduction, greatly improving the signal quality. Data were taken using $P_{l}$= 1 mW, $v_{f}$= 0.25 mm s^-1^, $T_{f}$= 1 s, and $N_{f}$= 1, planar scanning across (37 × 40) µm^2^ with 1µm steps. Spectral range 300 – 4500 cm^-1^, spectral resolution 10 cm^-1^.


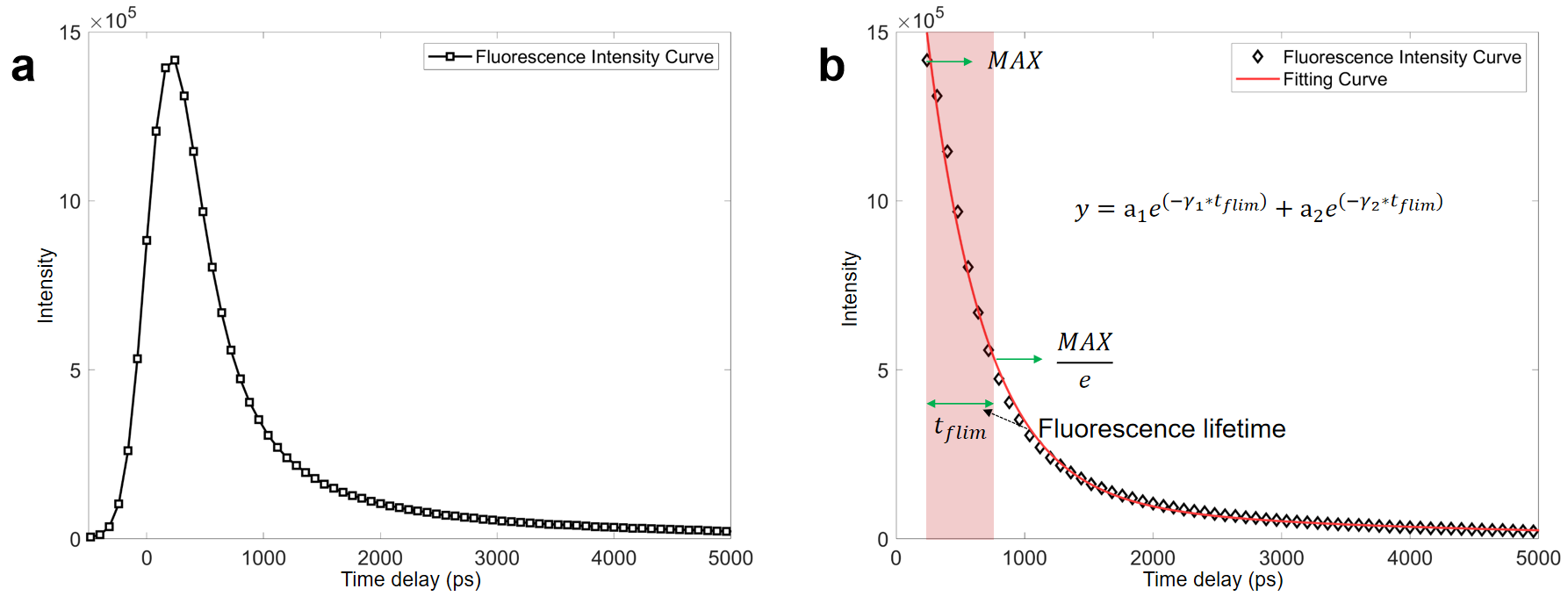
 **Fig. S8** The PS beads without R6G are used as an example to show how the fluorescence lifetime is calculated. **a** shows the time-resolved fluorescence intensity of the spheres. **b** shows the fitted curve of fluorescence lifetime. $a_{1}=2.34\times{10}^{6}$, $a_{2}=1.08\times{10}^{5}$ , $\gamma_{1}=2.2\times{10}^{-3}$ ps^-1^ and $\gamma_{2}=2.36\times{10}^{-4}$ ps^-1^. Fitting coefficient of determination $R^{2}=0.99$. $v_{f}$= 0.25 mm s^-1^, and $T_{f}$= 1s, summed over $N_{f}$= 10 repetitions.

Fig. S8 illustrates the extraction of the fluorescence lifetime from the time-resolved data. A fluorescence intensity curve is fitted. A total of more than 50 data points were fitted, with fit weights set to 5 for the first 30 data points (240 – 2640 ps), and 1 for the later ones. Find the time delay corresponding to the strongest point $MAX$ of fluorescence intensity, and then find the time delay corresponding to point ${MAX}/e$ (Obtain the exact time delay from the fitted curve). Calculate its relative time delay as $t_{flim}$.


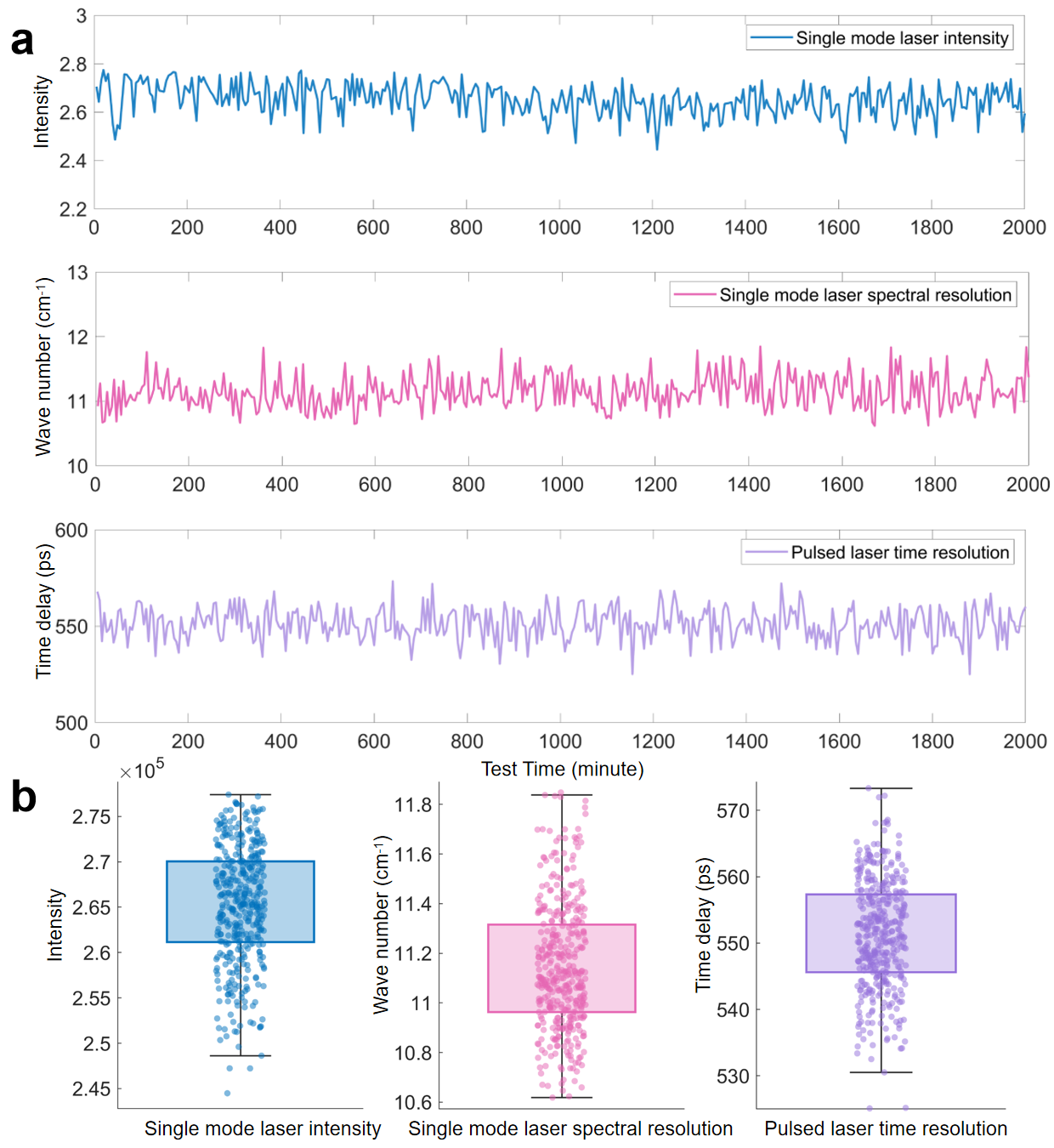


**Fig. S9** Instrument stability analysis over 33 hours duration. ​​**a**​​ Measured single mode laser peak intensity, spectral width, and pulsed laser temporal width. ​​**b**​ Statistical analysis using a data box plot. $v_{f}$= 0.25 mm s^-1^,$T_{f}$= 1 s, $N_{f}$=1, spectral resolution 10 cm^-1^. Data acquisition used a ​​5-minute interval​​ between single acquired spectra, with a total of 400 spectra acquired​​​.

Fig. S9 summarizes the instrument stability assessment, evaluating three key performance metrics: (1) spectral intensity, (2) spectral resolution, and (3) time resolution. Stability tests conducted with a single-mode laser yielded mean values of 265,437 ± 6,490 (Coefficient of Variation (CV) = 2.4%) for the peak intensity and 11.14 ± 0.25 cm⁻¹ (CV = 2.2%) for spectral width. Measurements using a pulsed laser system demonstrated a temporal resolution of 551.2 ± 8.2 ps (CV = 1.5%). The consistently low coefficient of variation (< 3%) across the measured parameters confirms the instrument's stability, satisfying the rigorous demands of precision optical spectroscopy.


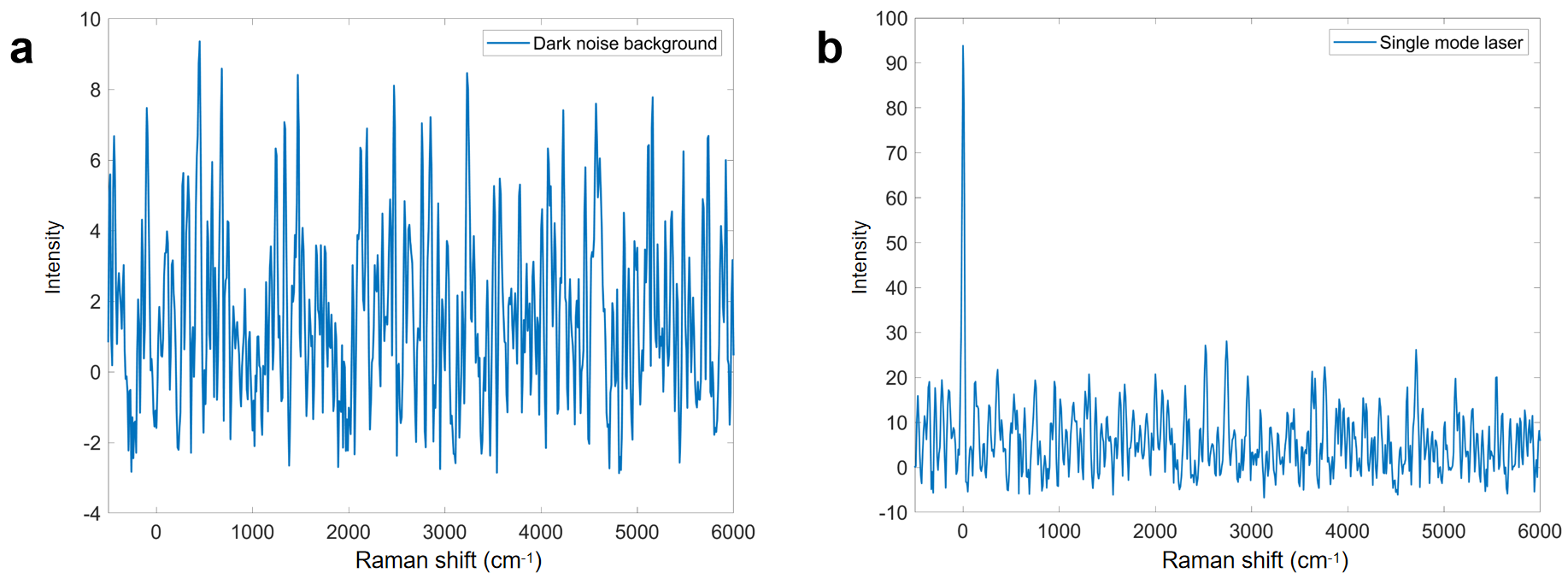


**Fig. S10** Instrument minimum detection limit scan test. **a** Dark noise background of instrument in darkroom. Photon counting rate 107 counts s^-1^. **b** Single mode laser spectrum at ultra-low counting rate. Photon counting rate 774 counts s^-1^. $v_{f}$= 0.25 mm s^-1^,$T_{f}$= 1 s, $N_{f}$=1, spectral resolution 10 cm^-1^. Shown is the spectral range -500 to 6000 cm^-1^.

Fig. S10 shows the instrument's single-mode laser spectral line detection capability under low count rate conditions. Under optical darkroom conditions, the system exhibits an ultralow dark count rate of 107 counts per second. When testing with a single-mode laser source attenuated to 0.1% transmission and coupled through a minimized diaphragm aperture, the photon count rate was 774 counts per second. The instrument clearly detected the spectral line of the single-mode laser under these conditions, confirming its exceptionally low detection limit. We note that this limit is determined by the dark noise of the APD detectors used, and lower dark noise versions are available at a higher cost.


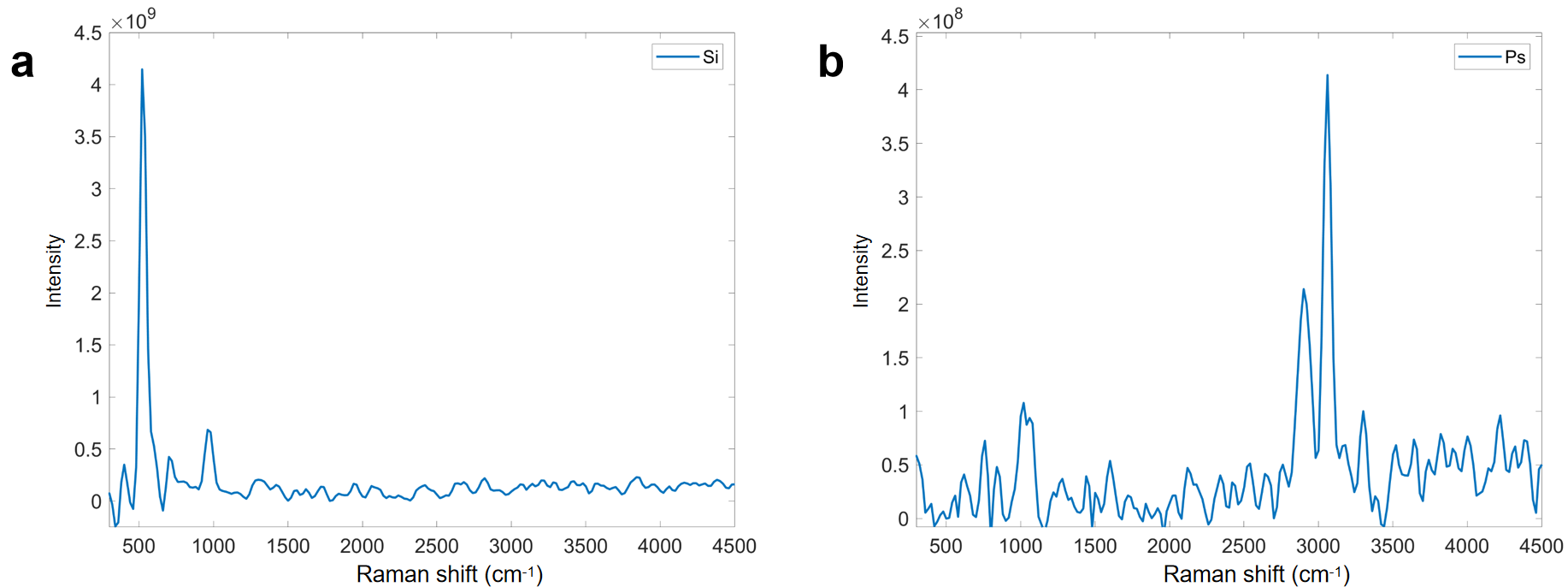


**Fig. S11** Spectra obtained using a shorter total optical path for faster scanning. **a** silicon (Si). **b** Polystyrene (PS) sheet. $v_{f}$= 0.25 mm s^-1^,$T_{f}$= 0.5 s, $N_{f}$=10, spectral resolution 20 cm^-1^. Shown is the spectral range 300 to 4500 cm^-1^, pulsed laser power 2.5 mW.

Fig. S11 shows the shortening of the total optical range difference to obtain a shorter scanning time at the same scanning speed. The spectral resolution is doubled due to halving the total optical range difference. However, the Raman spectrum is still clearly visible with a good signal-to-noise ratio, The intrinsic Raman linewidth of silicon is around 4  cm^-1^ at room temperature, so that the measured linewidth is resolution limited.
